# How often do awake craniotomies in children and adolescents lead to panic and worry?

**DOI:** 10.1007/s00381-023-06117-6

**Published:** 2023-08-23

**Authors:** K. Daniel O’Leary, Anastasia J. Philippopoulos, Alexis Koslofsky, Yashna Ahmed

**Affiliations:** 1 Department of Psychology, Stony Brook University, Stony Brook, NY USA; 2 School of Medicine, Department of Psychiatry &, Stony Brook University, Behavioral Health, RenaissanceStony Brook, NY USA

**Keywords:** Awake craniotomy, Pediatric brain surgery, Psychological sequelae

## Abstract

**Purpose:**

Awake craniotomy (AC) is the treatment of choice for the resection of brain tumors within eloquent brain regions for adults, but not much is known about its psychological impact on children and adolescents. Patient immaturity and difficulty in cooperating during surgery could result in psychological sequelae postoperatively, such as anxiety, panic, and worry.

**Methods:**

In this review, we examined eight studies assessing AC performed on patients under 18 years of age (*N* = 85), noting exclusion criteria, interventions used, and psychological assessments implemented.

**Results:**

Initial assessments of cognitive functioning and maturity were conducted primarily to determine patient eligibility for AC instead of an age restriction. No standardized interventions were used to minimize anxiety associated with AC. Interventions ranged from almost nothing specified to exposure to videos of the operating room, hypnosis, repeated meetings with psychologists and speech therapists, extensive meetings with the surgery team, and thorough exposure to the operating room theater. With a few exceptions, there were no standardized pre- and post-AC assessments of psychological sequelae. Qualitative evaluations indicated that most children and adolescents tolerated AC well, but one study indicated detrimental effects on school attendance postoperatively.

**Conclusion:**

Given that most AC teams have a psychologist, it seems desirable to have pre- and post-AC psychological assessments using standardized measures of anxiety, trauma, and worry, as well as measures tailored to AC, such as time to return to school, worry about MRIs following surgery, and self-assessment of post-surgery functioning. In short, comprehensive psychological assessment of AC patients is clearly needed.

## How often do awake craniotomies in children and adolescents lead to trauma and worry

Awake craniotomy (AC) was introduced during the 1920s by renowned neurosurgeon, Dr. Wilder Penfield, to treat intractable epilepsy in adults in the USA. The revolutionary surgery aimed to preserve patient neurological functioning and monitor motor and somatosensory brain areas by keeping a patient conscious intraoperatively to guide surgeons in tissue removal [1, 2]. Although removing minimal tissue increased the chance of lesion recurrence, doing so was shown to effectively reduce cognitive deficits and ultimately improve the patient’s quality of life postoperatively [3, 4]. Penfield further advanced the field of neurosurgery by outlining the role of anesthetic techniques in monitoring a patient’s state of consciousness and pain level during a procedure. Using anesthetics allowed him to achieve optimal brain mapping while preserving patient comfort [5].

Since created, AC has evolved into a standard procedure for brain surgery in most hospitals and surgical centers worldwide. Modern AC is used to allow resection of lesions near eloquent cortical areas. Sometimes electrophysiological recordings are used for additional mapping during surgery [3]. Comparatively, few children undergo the procedure, so research concerning the benefits and risks of pediatric AC is lacking substantially [6]. As noted by Balogun et al. [7] some surgeons were hesitant to perform AC on patients less than 15 years of age due to differences in their brain structure. Children and adolescents have a larger number of small and unmyelinated fibers that require a higher charge density to evoke an adequate response to neurostimulation, which could harm them [7, 8]. However, as noted herein, children have been successfully operated on with AC who are as young as 7 years, and children under 15 years of age are eligible for AC in certain centers if they pass neurological, psychological, and behavior evaluations prior to the surgery [8].

Mishra et al. published a review of AC in children that provided a detailed description of the children’s preoperative team visit, preoperative workup, positioning of the child, and sedation protocol. However, they did not evaluate the influence of AC on children’s psychological sequelae caused by or related to the procedure [9].

The purpose of this manuscript is to evaluate the psychological consequences of AC in children. We examined any psychological sequelae prompted by the surgery to determine whether a patient’s age or other factors should influence AC eligibility. We reviewed published, peer-reviewed articles that addressed patients’ psychological functioning pre- and postoperatively, focusing particularly on worry, anxiety, and trauma symptoms, and their influence on the child’s overall functioning. We also examined psychological evaluations completed on pediatric patients with directed attention to the following:

1. Were there standardized assessments of psychological functioning pre- and postoperatively?
2. Did the assessment go beyond standardized measures of generalized anxiety and panic to address the patient’s reaction to the surgery itself?
3. Were psychological interventions implemented, such as hypnosis, used to minimize the psychological impact of AC?

## Methods

Eight studies were collected regarding AC performed on children and adolescents under 18 years of age. The following key words and phrases were searched using Google Scholar, PsycINFO, and Academic Search Complete: *glioblastoma in children*, *awake craniotomy surgery*, *awake brain surgery*, *awake craniotomy procedure*, *awake craniotomy in children*, *awake craniotomy in adults*, *psychological sequelae of awake craniotomy in children*, *psychological sequelae of awake craniotomy in adults*, *panic*, *anxiety caused by awake craniotomy*, *and psychological interventions during awake craniotomy*. Information about the effects of AC in children and adolescents is presented in Table 1 and discussed in the main body of the paper. The table outlines the sedation protocol used, characteristics of patients in the sample, type of psychological assessments implemented, whether an intervention was used to reduce anxiety of the patient, and the conclusion reached about psychological sequelae prompted by AC.

**Table 1:** Effects of AC in children and adolescents

Source	Sedation protocol	Sample characteristics	Psychological assessments	Intervention type	Psychological sequelae	Conclusions
Delion et al. (2015) France	Fully awake	Children (*n* = 6) between 11 and 16 years old with supratentorial brain lesions (tumor and cavernoma)	Neuropsychological evaluation pre- and postoperatively based on the Boston Diagnosis Aphasia Examination. Follow-up evaluation timeline ranged from 1 week to 74 months postoperatively (*M* = 24.3 months). A French-adapted object denomination scale, Denomination Orale 80 (DO-80) was used preoperatively	Hypnosis conditioning, psychological support, introduction of the child to another child who has previously undergone AC	Depressive symptoms found in a patient postoperatively who had a preexisting anxious personality	1. Hypnosis conditioning was thought to contribute to reduced anxiety levels in patients 2. Preoperative hypnosis preparation for children undergoing AC was thought to lead to the best psychological outcome
García-Tejedor et al. (2020) Canada	Asleep-awake-asleep (96.7%) Conscious sedation (3.3%)	Children between 7 and 17 years old (*n* = 28, *M* = 14) primarily diagnosed with tumor, epilepsy, or arterio-venous malformation	Preoperative neurological evaluation to assess intellectual abilities, and speech, motor, and visual function	Distribution of detailed information about the surgery itself, potential complications, and intraoperative tasks, with assurance of their understanding	Psychological sequelae were not assessed	1. It is feasible to conduct AC in children 2. To maximize success, patients should be properly evaluated and prepared for the surgery, and should work closely with their surgical team 3. Research on the best anesthetic approach and drug combinations for AC in children is lacking
Huguet et al. (2019) France	Asleep-awake-asleep	Children between 9.4 and 17.6 years old (*n* = 17, *M* = 14.8) with a supratentorial lesion in a functional brain area	Psychological criteria including patients’ maturity, motivation, existing psychopathologies, confidence, understanding, and support were assessed preoperatively Identical assessments were conducted postoperatively at 3, 6, and 12-month follow-ups	Psychological preparation of the patient and their family for the procedure, including knowledge about the process, speaking with members of the surgical team, and intraoperative task training	Positive experiences were described in some, but others noted persisting disruptive feelings One patient that viewed the experience as negative experienced persistent depressive thoughts and PANIC symptoms such as anger, discomfort, and expressed feelings of emotional pain postoperatively	1. Age is not a reliable measure of a child’s maturity or preparedness for AC, so assigning an age limit to the procedure would be difficult 2. Psychological preparation for the procedure, as well as support, are important to the children’s well-being. Psychological preparation of children for AC should be mandatory 3. AC could potentially lead to PANIC or pediatric medical traumatic stress syndrome (PMTS) in children due to factors relating to fear surrounding their diagnosis, so it is important that children are thoroughly evaluated prior to undergoing surgery
Klimek et al. (2004) Netherlands	Protocol was unspecified, but context alludes to asleep-awake-asleep sedation protocol	Nine-year-old child (*n* = 1) with a recurrent high-grade glioblastoma in the left temporo-parietal region	Preoperative psychiatric evaluation to assess eligibility; criteria was unspecified Assessed 1 and 4 days postoperatively; criteria was unspecified	Psychological preparation, including training for intraoperative tasks and a detailed explanation of all procedure details given to the patient and their parent	No psychological sequelae noted	1. AC is safe in children with mild sedation if they are properly evaluated preoperatively and prepared for the procedure. Age restrictions for AC should not be upheld
Labuschagne et al. (2020) South Africa	Asleep-awake-asleep	11-year-old female with a dysembryoplastic neuroepithelial tumor (DNET) in the right motor cortex	Preoperative eligibility assessment of the child by surgical team Standard 6-week postoperative follow-up	Simulated surgical experience, support by speech therapist, rehearsal of intraoperative stress coping skills, explanation of procedure by surgical team	No pain or anxiety reported intraoperatively Patient noted that the simulated surgical experience prior adequately prepared her, which reduced her anxiety entering the procedure No negative emotions related to the surgery were reported	1. Individual child developmental markers should be used to assess a child’s eligibility for AC instead of age. A child’s readiness should be evaluated thoroughly before the procedure 2. Implementing a simulated surgical experience was helpful in minimizing psychological sequelae experienced by the child
Lohkamp et al. (2020) France	Asleep-awake-asleep	Children between 9.4 and 17.6 years (*n* = 18, *M* = 14.8) with brain lesions in or near eloquent brain areas, most being intrinsic tumors of glial origin -constant interactions with a neuropsychologist during surgery	Preoperative clinical evaluation, postoperative evaluation within 24 h of surgery and at 3, 6, and 12-month follow-ups Pre- and postoperative evaluations involved MR imaging, psychological and neuropsychological assessments, and a standard neurological function assessment	Surgery preparation (i.e., training for intraoperative tasks)— intensity of preparation varied depending on the patient’s individual needs	PANIC symptoms were present in 1 patient postoperatively	1. AC can be performed safely in children with the same benefits that are present for adult patients undergoing AC. Surgery tolerance was shown to be high in this young sample 2. AC should be considered for patients under 9 years of age with careful evaluation
Riquin et al. (2017a) France	Fully awake	Children (*n* = 7) between 8 and 16 years old with lesions in eloquent brain areas	Neuropsychological evaluation with child language therapist preoperatively, during the surgery, immediately after surgery, and at 3 and 6 months postoperatively. Child psychiatrists evaluated patients for psychiatric sequelae preoperatively, immediately after, and 3 months after surgery	Hypnosis conditioning for 6 patients	Child without hypnosis conditioning showed low levels of depression postoperatively One patient displayed depressive encephalopathic features temporarily after surgery that related to fear of relapse	1. Mental suffering of the patient with depressive symptoms seemed to be linked to neurologic symptoms from the lesion and news of the disease rather than the surgery 2. Anxiety is always present preoperatively but further studies are needed to examine this relationship 3. AC is well tolerated by children in this sample, but a child’s perception of the surgery is yet to be explored. Research evaluating the influence of AC on development of psychological sequelae in children is lacking
Riquin et al. (2017b) France	Protocol unspecified	8-year-old male with a left paramedian frontal tumor	Preoperative evaluation and evaluations immediately and 3-months postoperatively included the General PedsQL, Global Assessment of Functioning (GAF), and Hamilton Anxiety Rating Scale Neuropsychological evaluations using the Wechsler Intelligence Scale for Children and other cognitive inventories were conducted three times preoperatively, once immediately after surgery, and 1 year postoperatively	Hypnosis conditioning, exposure of the patient to the operating room	GAF scores decreased from 91 to 75, reflecting light impairment School Functioning decreased from 95 to 65 Hamilton Anxiety Scale Scores increased from 2 to 12 from pre- to 3 months post-surgery, reflecting mild severity Emotional Functioning decreased from 100 to 40 No PANIC symptoms were exhibited 1-year postoperatively	1. AC is feasible in young children 2. Making the patient and their family comfortable with the operating environment and procedure were important. They note that pictures, videos, visiting the operating room, and getting to know the medical team were helpful in particular 3. Hypnosis was a helpful intervention

## Results

### Number of patients in each study

Three papers examined single-case studies, two studies included 6–7 children, two included 17–18, and one included 28 patients. Thus, the small sample size per study generally prevented parametric analyses of predictors of response to treatments. Nevertheless, certain conclusions were clearly provided by the authors.

### Sedation protocols implemented

Two types of sedation protocols were implemented: (a) the fully awake (FA) protocol, in which patients remained fully conscious throughout the entire procedure, and (b) the asleep-awake-asleep (AsAAs) protocol, in which patients were sedated upon opening and closing of the dura and skull but awakened during lesion resection. The AsAAs protocol is preferred by many centers due to patients’ experience of discomfort, pain, vibrations, and loud noise during the opening and closure of the cranium [10]. Bianco et al. note that the Maggiore della Carità University Hospital in Novara, Italy, has performed 27 brain surgeries using FA protocol since 2015, and there has been only one case of evident psychological strain during the closure phase of a long-lasting surgery [11]. As a result of this case, the hospital implements AsAAs protocol unless the surgery is brief and simple, the patient is ineligible for general anesthesia, or an intraoperative awakening is expected [11]. The FA protocol might be chosen in resource-limited countries as well [11]. Out of the eight studies reviewed, two primarily adhered to the FA protocol, four adhered to the AsAAs protocol, and two did not specify the sedation protocol implemented.

### Exclusion criteria

The appropriateness of AC was often determined by a child’s maturity and cognitive abilities, which were generally measured by IQ, executive functioning, academic capabilities, language and memory skills, and manual dexterity [12]. In Alcaraz García-Tejedor et al., it was not specified what percent of possible candidates were excluded based on the above criteria [12]. One of the 28 children had to have the operation under general anesthesia due to severe intraoperative agitation. The pediatric psychiatrist did not contraindicate any child for AC in the study completed by Delion et al. [6]. Huguet et al. proposed AC for 18 patients and after psychological evaluation, AC was performed on 17 patients [13]. Riquin et al. included 7 patients who had AC, two of which had high levels of preoperative anxiety and were still included. The surgery took place without difficulty [14].

### Interventions used to reduce anxiety of the patients

The study completed by Delion et al. involved 6 patients aged 11–17 years (*Mean* = 13.67), four of whom were teenagers [6]. The patients received hypnosis conditioning, neuropsychological examination by a speech therapist, psychiatric evaluation, and neuropsychiatric follow-ups in all cases. Members of the surgical team showed patients videos of the operating room preoperatively and introduced the children to another child their age who had undergone AC. Hypnosis conditioning was completed by the anesthetic team.

Klimek et al. illustrated the use of AC in a 9-year-old boy, which is presumed to be the first publication defying Pasquet’s (1954) position that “uncooperative adults and children under 10 years will not tolerate the application of local anesthesia, scalp incision and craniotomy” [15, 16]. The patient was thoroughly exposed preoperatively to the procedures used in the operating room. There was no discussion of psychological procedures used to minimize anxiety pre- and postoperatively, or information provided on psychological assessments used.

Huguet et al. comprehensively described their preoperative evaluation and preparation of patients [13]. All children had neurological exams and MRIs. Patients whose MRIs confirmed a supratentorial lesion in a functional area were candidates for AC. Psychological assessment and preparation were conducted by a certified psychologist across several meetings with the patient to ensure that the patient and family understood AC and its psychological implications. The number of preoperative meetings was determined on a case-by-case basis, but the meetings addressed anticipated emotions and concerns. During the surgery, a neuropsychologist had constant interactions with the patient.

In the study completed by Alcaraz García-Tejedor et al., more information was provided about the anesthetic and surgical procedures than about psychological interventions [12]. They expressed that an assessment was conducted by a neuropsychologist that focused on neuropsychological deficits, language deficits, and presenting symptoms. They also noted that pictures and videos were used to help explain AC to the children.

In addressing the needs of an 11-year-old female with significant distractibility and inability to follow commands, Labuschagne et al. developed a detailed hospital theater experience for this child that has now become standard practice at the Department of Surgery at the University of Witwatersrand, South Africa [17]. The intervention involved having the child experience as much as possible of the actual surgery procedures before undergoing the surgery. As they stated, “the patient was dressed in theatre attire and brought into the theatre on a theatre trolley. She was then transferred onto the theatre bed and positioned in the same manner as she would be for the actual surgery. Her head was placed on a horseshoe headrest, and she was made to lie in a semi-lateral position, as required for the surgery. A blood pressure cuff, pulse oximeter, nasal cannula with oxygen flow, and calf pumps were applied. She was then draped precisely as she would have been for the procedure. Theatre lighting was set as it would be for the surgical case.” (p. 1) [17]. In short, the simulated theater experience allowed the hospital staff to induce typical stress provoked by the procedure. Doing so allowed the surgical team to desensitize the patient to the hospital procedures while assessing her coping skills. The authors also emphasized the development of a strong bond with a speech therapist during the simulation. This relationship was presumably important in the child having no postoperative psychological symptoms or anxiety.

### Standardized measures used to assess anxiety and panic symptoms

As seen in Table 1, the psychological assessment instruments used to evaluate pre- and postoperative anxiety generally were not standardized. The exceptions were present in the case study by Riquin et al., in which the Hamilton Anxiety Rating Scale [18], Global Assessment of Functioning (GAF) [19], and the Peds Quality of Life questionnaire (PedsQL) [20] were used to assess the overall functioning of an 8-year-old girl who had a resection of an intracerebral tumor [21]. This patient was a participant in a larger study by Riquin et al., but these measures were not reported in that paper [14].

In other studies, researchers implemented assessments of their own or briefly described the assessments completed. For instance, Huguet et al. provided detailed descriptions of the psychological outcomes of 17 children who underwent AC [13]. While standardized measures of anxiety and panic were not used, the detailed qualitative information provided about the psychological functioning of 8 of the 17 children was useful. One child was excluded preoperatively because of a diagnosis of OCD and recurrent depression, as psychological conditions were considered contraindications for AC by that team. Limited eligibility was seen in one patient who suffered from depression early in life and another who had pervasive developmental disorder. Two of the 17 AC patients died from tumor progression, ultimately preventing surgical staff from evaluating their emotional experience long-term. However, immediately after surgery, they did not present with negative emotions. Long-term follow-ups were available for eight of the 17 children in the form of psychological evaluations and were conducted at 3, 6, and 12 months postoperatively. Each patient met the surgeon, neurologist, psychologist, and neuropsychologist during these timepoints. The overall evaluation was positive for six children. Two children were content with the experience, but they persistently experienced disrupting feelings associated with it. One patient claimed that AC evoked fear and was painful, and the patient exhibited partial panic symptoms and persisting depressive thoughts postoperatively. This report should be respected for its frank and direct concern about the psychological effects of AC on children and adolescents since they state that AC carries a risk of its own psychological morbidity. They further note that AC needs to be considered as a potential traumatizing event and a possible cause for panic.

The case study completed by Klimek et al. indicates that the child was evaluated by a child psychologist to assess eligibility for the procedure [15]. Other psychological components, such as nightmares, levels of cooperation, endurance, emotional reactions, and ability to concentrate on difficult tasks were noted solely for eligibility purposes [15]. No standardized measures of anxiety or panic were used to assess the 9-year-old pre- and postoperatively for the resection of a glioblastoma.

Some detail is provided on the 6 cases reported in the Delion et al. study, as the authors provided some very useful clinical data, such as time to return to school and the patient’s academic performance [6]. We believe that information on time to return to school, school grades, and ability to return to extracurricular activities would be useful to report. Authors of this study aided readers by outlining the types of assessment used to evaluate their patients, and five of the six were evaluated 3 months postoperatively. Two patients returned to school 6 months post-surgery, and one returned to school full-time 3 months post-surgery; one had not returned to school 6 months post-surgery and was being taught in a hospital school. In one case, post-secondary school was completed and the individual entered medical school. In sum, the surgeries were associated with absence from school for a number of months, and some verbal problems persisted. Reports of time to return to school and to various activities would be useful to note in any AC outcomes, following the lead of Delion et al. [6].

Alcaraz García-Tejedor et al. focused more on neurological evaluations and physical observations than psychological components of the procedure [12]. Their primary outcome measure was feasibility of AC, defined as the ability to complete the procedure without conversion to general anesthesia. Median age of the children was 14, and they showed that AC was feasible in 29 of the 30 cases, with only one patient converted to general anesthesia. No patients were excluded. New neurological deficits occurred in 20% of the patients, but the deficits were transient. The researchers did not assess the psychological impact of the AC, but there were no explicit complaints of emotional distress at a postoperative follow-up.

## Discussion

There are no systematic evaluations of the psychological interventions used to minimize anxiety in children or adolescents undergoing AC, even though almost all of the studies reviewed herein emphasize the need for such interventions. Given the gravity of possible operation-induced trauma, it is understandable that control groups lacking an intervention to reduce anxiety were avoided. However, alternative interventions used to minimize trauma and anxiety should be evaluated in the future. Interventions that seemed to improve patient outcomes include hypnosis conditioning, rapport building, thorough exposure to the operating room, and surgical team preoperatively, introducing the patient to someone their age who has undergone a successful AC, showing the patient a video of an operation or recovery preoperatively, and simulating the operating theater experience preoperatively. These options could be evaluated as anxiety intervention procedures using standard experimental vs. control comparisons. Neuropsychologists were nearly always involved in the overall surgical intervention across all countries examined. Accordingly, they could help organize psychological evaluations and systematically collect preoperative, postoperative, and follow-up data.

The specifics of a child’s tumor will determine the extent to which psychological interventions can be used to minimize anxiety. If a patient presents with a large or a high-grade tumor, there will be little time for psychological preparation work as surgery would be ideally scheduled within a few days to relieve intracranial pressure and reduce the risk of further tumor growth. In short, there is a need to balance risk of anxiety and psychological interventions with the need for immediate surgery. Nonetheless, variations of the hospital theater experience at the University of Witwatersrand described above could presumably be implemented a day or two before surgery.

While most authors of the studies reviewed herein emphasize the need to conduct psychological evaluations of the children and adolescents who are potential candidates for AC, the percent of children or adolescents screened out for AC often was not specified. Mishra et al. noted specific contraindications that led them to conclude absolutely that children or adolescents should not have AC [9]. Those absolute contraindications were as follows: patient/parental refusal to consent; uncooperative child; mental retardation; agitated child; profound dysphasia or language problem; learning/cognitive disabilities. Future research should include specification of the number of patients excluded and exclusion criteria to help researchers evaluate whether more children could successfully undergo AC than was originally presumed.

The literature observed lacks several details that would likely improve psychological evaluations of children and adolescents for AC. For instance, there was no explicit mention of whether patients’ tumors were cancerous and whether patients had undergone AC in the past. In addition, repeated unsuccessful surgeries could potentially make patients feel hopeless about their recovery process and the results of the procedure, which could exacerbate psychological sequelae prompted by AC. It is also recommended that all psychological evaluations take patient concerns about MRIs into consideration since many individuals expressed anxiety over completing MRI scans and may respond negatively to MRI results. An overall evaluation of the patient’s quality of life and time needed to return to varied important activities such as school (part-time; full-time), exercise (modified; non-modified), and other extracurricular activities should also be implemented. The minutes spent in each activity as well as the total minutes spent in preoperative preparation could be correlated with anxiety and worry scales. Lastly, documenting the type and extent of preoperative interventions used to minimize anxiety in the children and adolescents would be beneficial as well. Pre- and post-standardized measures of psychological functioning have not been used routinely, and if used, could contribute significantly to the literature on psychological functioning of the children and adolescents undergoing AC. Not only would it be helpful to have standardized measures of anxiety, panic, and worry for children and adolescents undergoing AC, but also it would be helpful to have measures that specifically address memories of the brain operation and post-surgery functioning. We suggest the Modified Penn State Worry Questionnaire for Children and Adolescent [22], the Youth Anxiety Measure for DSM-V [23], or the Screen for Child Anxiety Related Emotional Disorders [24]. The Modified Penn State Worry Questionnaire for Children (PSWQ-C) is a 14-item self-report questionnaire to assess worry in children and adolescents aged seven to seventeen. It measures the tendency of youth to engage in excessive, generalized, and uncontrollable worry. Examples include “My worries really bother me,” and “I know I should not worry but I just can’t help it.” The PSWQ-C is readable at the second-grade level and it has excellent internal consistency with an alpha of 0.89. The Youth Anxiety Measure for DSM-V has 28 items in the first part of the measure to assess the major anxiety disorders of the DSM-V such as separation anxiety, social anxiety, and panic disorder; the second part containing 22 items is used to measure specific phobias and agoraphobia. Cronbach’s alpha for the total scale was excellent for both clinical and non-clinical samples, 0.93 and 0.92., and parent child agreement in a clinical sample was quite good, 0.69 for major anxiety disorders, and 0.70 for phobias. The Screen for Child Anxiety and Related Disorders is a 41-item scale that is composed of the following five factors: panic/somatic symptoms, generalized anxiety disorder, separation anxiety disorder, social anxiety disorder, and school avoidance. The alphas were above 0.78 reflecting good internal consistency. The parent–child correlation for the total anxiety scale was 0.32 with a much higher correlation for those individuals older than 12 (0.43) compared to those children aged 9–12 years (0.03). Given the desirability to assess worry in children and adolescents undergoing AC, the Penn State Worry Questionnaire for Children seems very appropriate for such individuals, and it seems appropriate to also use the Youth Anxiety Measure as it measures DSM-V anxiety disorders, and it has much higher parent–child agreement than the Screen for Child Anxiety and Related Disorders. However, we believe it is crucial to conduct more specific assessments of content more related to the AC, and we provided specific scales to measure (1) the amount of intervention time used to prevent anxiety associated with AC, (2) anxiety regarding memories of the brain operation assessed 1 week after the AC, e.g., assessing anxiety when I first heard I was going to have a brain operation, and assessing anxiety when I first learned I would be awake during surgery, (3) assessment of specific worries about tumor recurrence, seizures, side effects of medications, etc., 1 week and 3 months after surgery, (4) measures of functioning 1 month post-surgery, e.g., ability to speak clearly, ability to use read for long periods of time, (5) assessment of number of weeks before return to school part-time and full-time, and weeks before return to physical education and competitive sports. Such assessment measures appear below in Tables 2, 3, 4, 5, and 6. The rating scale measures are included in Tables 2, 3, 4, 5, and 6 to assist researchers in having specific targeted measures to evaluate the impact of AC on children and adolescents. These measures are proposed to allow a clinician to monitor progress in a concrete quantitative manner of anxiety of memories of AC, specific worries of tumor recurrence and side effects of anti-seizure mediations, verbal and attention functioning post- surgery, and weeks before return to school and athletic events. If all children receiving AC in a large hospital were evaluated using such targeted measures, the aggregated data could be used to help a clinician provide individual feedback to parents and children on how an individual patient fared compared to large numbers of other children receiving AC. Finally, it seems important for both clinicians and researchers to document the type of intervention(s) used to reduce postoperative anxiety and worry, and the amount of time used to implement the intervention(s) to allow for subsequent intervention comparison and evaluation.

**Table 2:** Intervention type and intervention time spent in hours or minutes

Type of intervention	Hours	Minutes
Hypnosis		
Saw video of child or adolescent who had awake craniotomy		
Interacted personally with child or adolescent who had awake craniotomy		
Viewed video of operating room		
Met with neuropsychologist before operation		
Met with surgeon before operation		
Met with anesthesiologist before operation

**Table 3:** Memories of my brain operation: assessed 1 week after my surgery

	Not anxious	A little anxious	Anxious	Quite anxious	Very anxious
1. When I first heard I needed to have a brain operation, I was…	0	1	2	3	4
2. When I first heard I might be awake during my brain surgery, I was…	0	1	2	3	4
3. When I was led into the operating room, I was…	0	1	2	3	4
4. When I first heard the noise of the drilling into my skull, I was…	0	1	2	3	4
5. When I heard discussions of the surgeon and neuropsychologist during the operation, I was…	0	1	2	3	4
06. When I woke up just after the operation was over, I was…	0	1	2	3	4
7. When I realized how my speech sounded when I first talked right after the operation, I was…	0	1	2	3	4
8. A week before my first MRI after my surgery, I was…	0	1	2	3	4
9. When I think about a possible recurrence of a tumor, I am…	0	1	2	3	4

**Table 4:** Worries about tumor: assessed 1 week and 3 months after surgery

	Never	Sometimes	Often	Very often
1. I worry about the possibility of a tumor recurrence	0	1	2	3
2. I worry about having additional MRIs	0	1	2	3
3. I worry that I will have to have another brain surgery	0	1	2	3
4. I worry that since they had to remove part of my brain that I will never be as good as I could have been if I did not have the brain surgery	0	1	2	3
5. I worry about seizures	0	1	2	3
6. I worry about the side effects of the medications I had to take after my surgery	0	1	2	3

**Table 5:** Measure of post-surgery functioning 1 month after the operation

	Not able	Partly able	Quite able	Fully able
1. I rate my ability to speak clearly one month after the operation as…	0	1	2	3
2. I rate my ability to use my arms or legs one month after the operation as…	0	1	2	3
3. I rate my ability to read and fully concentrate one month after the operation as…	0	1	2	3
4. I rate my ability to read for long periods of time now one month after my operation as…	0	1	2	3

**Table 6:** Return to school post-surgery: assessed 6 months after surgery

	Weeks after operation (if applicable)
I returned to school part-time…	
I returned to school full-time…	
I was able to participate physical education…	
I was able to participate in competitive sports (like my school or town soccer league)…	

Qualitative data reviewed herein indicated that most children and adolescents appear to tolerate AC well, but one study indicated detrimental effects on school attendance postoperatively. Additionally, there have been no studies with systematic evaluation of anxiety, worry, and PTSD post-surgery. With data from scales suggested herein with samples of children who had AC, as well as open-ended interviews to allow the children or adolescents to express any worries or concerns regarding the AC, one can conduct a systematic evaluation of the potential for anxiety and worry following AC.

## Conclusions

Returning to the three initial questions posed in the “Introduction” section of this paper, first we can unequivocally state that there were no standardized assessments of psychological functioning pre- and postoperatively. Second, in one study, the assessments did go beyond standardized measures of generalized anxiety and panic to address the patient’s reaction to the surgery itself as seen in the case of Delion’s measures of time to return to school and academic performance. Third, quite varied psychological interventions such as hypnosis and exposure to the operating room theater were used to minimize anxiety and worry in patients. Unfortunately, the interventions varied greatly and there was no way to systematically evaluate the effectiveness of the interventions to reduce anxiety and worry. It seems an opportune time to systematically evaluate the effectiveness of psychological interventions used in concert with AC, and standardized assessments of worry and DSM-V anxiety disorders are suggested along with measures designed to assess time spent in the psychological intervention used to reduce anxiety regarding AC, worries specifically related to AC, tumor recurrence, seizures, and weeks before return to academic and athletic functioning after AC.
